# Racial Disparities in Anesthesia Care: A Systematic Review of Pain Management and Patient Outcomes

**DOI:** 10.7759/cureus.68992

**Published:** 2024-09-09

**Authors:** Zachrieh Alhaj, Gengi Kleto, Zaid Almubaid, Mohammed Omar Almosa, Abdulkader Almosa, Sharif Mohamed

**Affiliations:** 1 Medicine, John Sealy School of Medicine, University of Texas Medical Branch, Galveston, USA; 2 Medicine, Edward Via College of Osteopathic Medicine, Monroe, USA; 3 Medicine, Texas Tech University, Lubbock, USA; 4 Anesthesiology, University of Texas Medical Branch, Galveston, USA

**Keywords:** • access to healthcare and health outcomes of vulnerable populations, anesthesia, inequity, pain, people of color, racial disparities

## Abstract

Racial disparities in healthcare are a prominent issue that needs to be addressed to improve the quality of care for all patients. There are several disparities and biases related to the perceived pain tolerance people of color (POC) patients have and their need for analgesics. These biases lead to inadequate pain management and decreased health outcomes. Our study aims to highlight these disparities and how they impact the care patients receive, specifically in the field of anesthesia. To conduct this study, a comprehensive systematic literature search was performed, articles were included and removed according to specific inclusion and exclusion criteria, and a systematic review was performed. Sixteen papers that met the inclusion and exclusion criteria were selected, and after data collection, correlations between POC and pain tolerance were assessed throughout the articles. The studies reviewed showed that there may be some correlation between racial background and perceived pain tolerance. While some studies found that racial disparities may negatively impact the care POC patients receive, others found that there was no correlation at all. Regardless, more studies need to be conducted to assess the factors influencing the treatment of POC in anesthesia.

## Introduction and background

Racial disparities in healthcare continue to be a pressing issue that needs to be addressed to provide the best care possible to people of varying ethnic backgrounds. The field of anesthesia is among many in medicine that has these disparities [1]. Within anesthesia, there is a tendency to view people of color (POC) as having higher pain tolerance, leading to inadequate pain management for POC patients [1]. General anesthesia, regional anesthesia, and sedation are all techniques used to reduce pain during operations; they have been found to reduce mortality rates, rates of infection, and time spent in a surgical setting. However, despite the multitude of benefits, these techniques are used less often in POC compared to their Caucasian counterparts [1]. In turn, this causes POC patients to experience increased recovery times, a higher risk of complications, and chronic pain issues [2]. Therefore, it is important to acknowledge and address these disparities to ensure that all patients receive adequate care, improve health outcomes, and reduce health inequities [2].

In our systematic review, we aim to bring to light the racial disparities found in the field of anesthesia, and its impact on pain management and patient care. We plan to compile literature that highlights the factors that led to these disparities and their role in health outcomes for POC patients. This review will provide an overview of the current state of racial disparities in anesthesia care and offer recommendations for future research to ensure equitable treatment for all patients.

## Review

Methods

A comprehensive systematic literature search was performed on June 12, 2024, in multiple databases, including Medline (Ovid interface), Scopus, and Cochrane Library. The search strings were produced by a reference librarian and another reference librarian. The search terms focused on POC, terms for specific racial and ethnic groups, racial bias or discrimination, pain, and anesthesia or anesthetics. Depending on the search functionality of each database, a combination of controlled vocabulary and keyword terms and truncation were utilized to find relevant literature on the topic. The search strings used for each database can be found in Appendix A.

**Figure 1:**
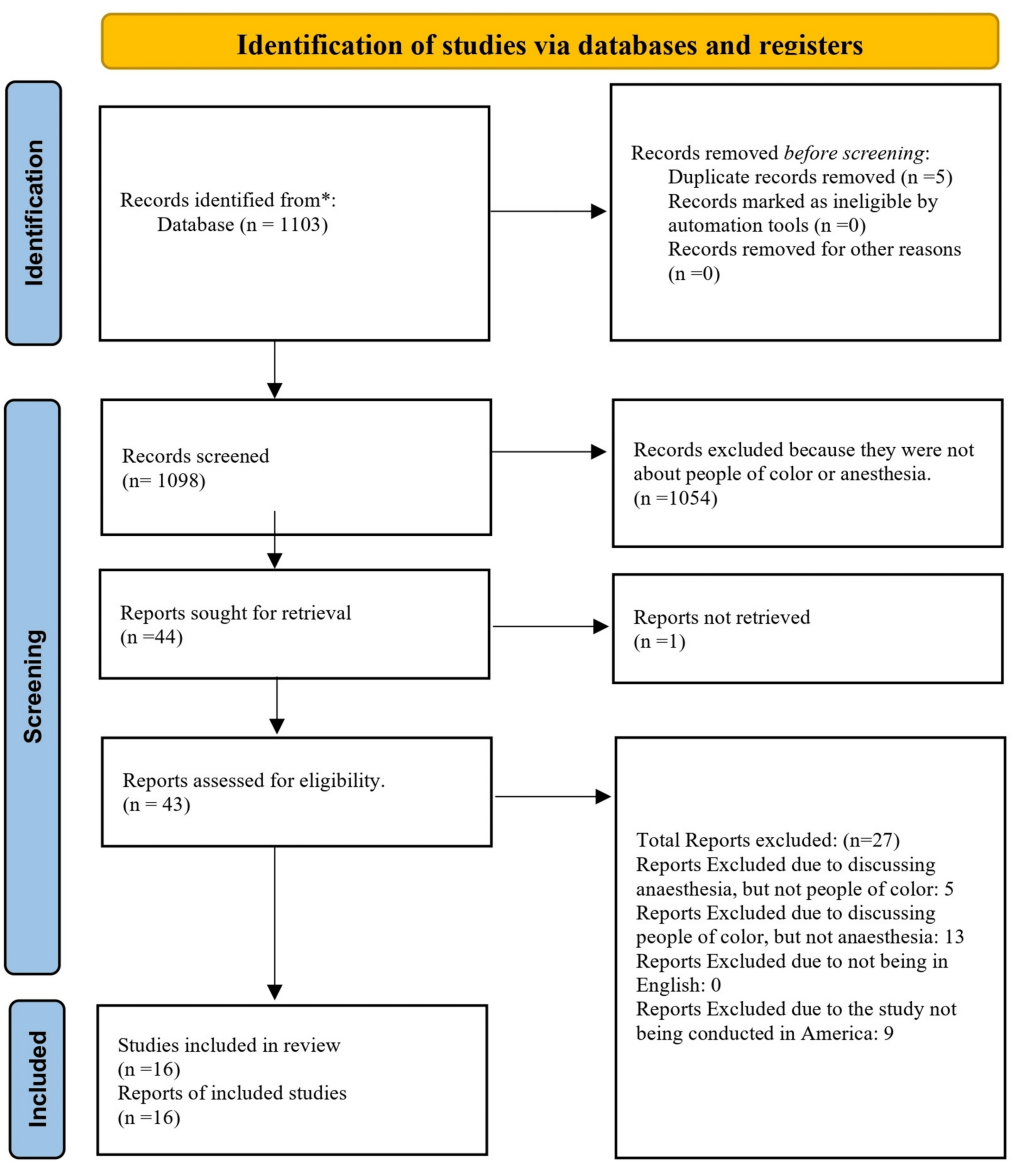
PRISMA model flowchart showing the selection of articles for review PRISMA, Preferred Reporting Items for Systematic reviews and Meta-Analyses

The search resulted in 1,103 papers, all subsequent literature was collected, and a total of five duplicates was removed, resulting in 1,098 articles eligible to be screened. The initial screening was based on the paper’s relevance to the topic. Papers were removed if they were not about the topic we were researching, anesthesia, and its impact on POC. After the initial review, 44 papers were left for review, one of which was not retrievable. The remaining 43 articles were reviewed and assessed based on our inclusion and exclusion criteria. Our inclusion criteria included any studies exploring the association between POC and anesthesia, written in English, and conducted within the United States. Exclusion criteria included papers that did not focus on POC (five articles excluded), did not focus on anesthesia (13 articles excluded), were not written in English (zero articles excluded), and were not produced in the United States (nine articles excluded). This review led to a final number of 16 papers that fit both the inclusion and exclusion criteria. The articles were consolidated, and limitations and potential future research were assessed, discussed, and recorded. Figure 1 shows a visual representation of the paper and data collection discussed here using the Preferred Reporting Items for Systematic reviews and Meta-Analyses (PRISMA) model. Table 1 shows a table summarizing the findings of each study included in this review.

**Table 1:** Comparative analysis of studies included in the review

Author and year	Study summary	Study design and population	Outcome measures
Baetzel et al. (2019) [3]	The aim of the study is to investigate and quantify unconscious racial bias in pediatric anesthesia care by examining disparities between Black and Caucasian children in the administration of inhalation induction, child life support, family presence during induction, and premedication with oral midazolam.	Investigative study on Black and Caucasian pediatric patient	Differences in anesthesia administration, child life support, family presence during induction, and premedication
Burton et al. (2021) [4]	The aim of the study is to evaluate the association between race and ethnicity and the use of neuraxial anesthesia for cesarean delivery in the United States and to assess any racial or ethnic disparities in perioperative anesthetic practices and postpartum adverse events.	Evaluation study on women undergoing cesarean delivery	Use of neuraxial anesthesia and perioperative and postoperative adverse events by race and ethnicity
Kahveci et al. (2024) [5]	The aim of this study is to examine whether racial disparities exist in immediate postoperative pain scores and intraoperative analgesic regimens in a single surgical cohort undergoing minimally invasive hysterectomy.	Retrospective cohort study on women undergoing minimally invasive hysterectomy	Postoperative pain scores and intraoperative analgesic regimens by race
Torres et al. (2022) [6]	The aim of this study is to investigate whether racial disparities in labor epidural analgesia at the hospital, which existed prior to the COVID-19 pandemic, were exacerbated during the pandemic.	Observational study on women receiving epidural before labor	Epidural analgesia use before and during the COVID-19 pandemic
Meier et al. (2021) [7]	The aim of this study is to evaluate whether the use of local anesthesia for open inguinal hernia repair varies according to patients’ race and ethnicity and to identify any racial or ethnic disparities in anesthesia modality, given the potential benefits of local anesthesia in reducing postoperative complications and operative time.	Evaluation study on patients undergoing inguinal hernia repair	Anesthesia modality for open inguinal hernia repair
Butwick et al. (2016) [8]	The aim of this study is to investigate whether racial and ethnic disparities exist in the mode of anesthesia (general versus neuraxial) for cesarean delivery and to examine how these associations are influenced by demographic and maternal factors, obstetric morbidities, and indications for cesarean delivery.	Investigative study on women undergoing cesarean delivery	Mode of anesthesia (general vs. neuraxial) used among different racial and ethnic groups and what influences these decisions
Toledo et al. (2012) [9]	The study aimed to analyze racial and ethnic disparities in neuraxial analgesia use and anticipated use among laboring Hispanic, African American, and Caucasian women and to evaluate sociodemographic, clinical, and decision-making predictors of these disparities.	Analysis study on Hispanic, African American, and Caucasian women undergoing labor	Use of neuraxial analgesia among different racial groups
Thomas et al. (2024) [10]	The aim of this study is to evaluate whether racial and ethnic disparities exist in the mode of anesthesia (general versus neuraxial) used for cesarean delivery and to determine if these disparities are influenced by demographic and maternal factors, obstetric morbidities, and indications for cesarean delivery.	Evaluation study on women undergoing cesarean delivery	Mode of anesthesia (general vs. neuraxial) used among different racial groups
Tangel et al. (2020) [11]	The aim of this study is to evaluate racial and ethnic disparities in severe maternal morbidity and administered anesthesia techniques, focusing on differences in outcomes and care among Black and White parturients.	Multi-state analysis study on Black and White parturients in the United States	Differences in outcomes and care
Silber et al. (2013) [12]	The aim of this study is to investigate whether BMI explains the previously observed racial differences in anesthesia procedure lengths between Black and White Medicare patients and to examine the relative contributions of surgical and anesthesia times to these differences.	Investigative study on Black and White Medicare patients	Procedure lengths and the influence of BMI
Mazzeffi et al. (2022) [13]	The aim of this study is to examine whether racial and ethnic disparities exist in the use of peripheral nerve blocks for postoperative analgesia in patients undergoing total mastectomy and to determine if PNBs are associated with reduced odds of major complications after the procedure.	Retrospective cohort study on patients undergoing total mastectomy	Associated complications in postoperative analgesia
Vingan et al. (2024) [14]	The aim of this study is to understand factors associated with the receipt of regional anesthesia blocks for mastectomy with immediate tissue expander reconstruction in a high-volume ambulatory surgery practice and to identify any patient-specific factors contributing to the decision to decline the procedure, despite a standardized approach offering preoperative nerve blocks to all eligible patients.	Observational study on patients undergoing mastectomy and tissue expander reconstruction	Receipt of regional anesthesia blocks
Wong et al. (2020) [15]	The aim of this study is to investigate the effect of race and subclinical elevations in blood pressure (prehypertension) on cutaneous sensory nerve-mediated and nitric oxide-dependent vasodilation, comparing normotensive and prehypertensive non-Hispanic Black and non-Hispanic White participants.	Investigative study on normotensive and prehypertensive non-Hispanic Black and non-Hispanic White patients	Cutaneous vasodilation responses
Guglielminotti et al. (2024) [16]	The aim of this study is to assess the association between structural racism and the use of labor neuraxial analgesia, analyzing differences in analgesia rates among non-Hispanic Black and White birthing people based on a structural racism index measured in the county of the delivery hospital.	Assessment study on non-Hispanic Black and non-Hispanic White birthing patients	Neuraxial analgesia and its association with structural racism index
Pace et al. (2023) [17]	The aim of this study is to explore the impact of age, racial, demographic, and psychosocial factors on the dosage of analgesia and maximum pain score experienced by patients during procedural abortion.	Retrospective cohort study on patients undergoing abortion	Analgesia dosage and maximum pain score
VanderBeek et al. (2006) [18]	The aim of this study is to identify the potential relationship between the use of pain medications and race in African American and Caucasian children undergoing forearm fracture reduction, examining factors such as fracture characteristics and patient demographics to determine if race influences the decision to use conscious sedation.	Investigative study on African American and Caucasian children undergoing forearm fracture reduction	Use of pain medications and conscious sedation

Results 

A total of 16 papers were included in the systematic review that fit the inclusion criteria. The studies all discussed different ways that racial disparities present, or do not present, in anesthesia via pain control and pre- and postoperative anesthesia administration. To allow for a broader understanding of our topic, we divided each of the 16 articles into three overarching themes: racial and ethnic disparities in anesthesia, the impact of structural racism on anesthesia patient care, and racial disparities in pain management. While each article we reviewed relates back to racial disparities in the field of anesthesia, the goal of the themes provided is to allow for a more focused understanding of each of the articles.

Theme 1: Racial and Ethnic Disparities in Anesthesia

Adultification of Black children in pediatric anesthesia: Retrospective data from 33,717 Caucasian and 3,901 Black children under 18 years of age were analyzed for unconscious racial bias in anesthesia care. Results showed that Black children aged 10-14 were 1.3 times more likely to receive mask induction compared to Caucasian children. However, Black children under 15 were significantly less likely to have a family member present during induction, and those under 5 were less likely to receive premedication with midazolam. Poor documentation prevented the analysis of child life consultation. The study concludes that disparities exist in anxiety mitigation strategies during anesthesia, possibly due to the adultification of Black children [3].

An update on racial and ethnic differences in neuraxial anesthesia for cesarean delivery: Of 12,876 participants, the study found that Black and American Indian or Alaska Native women had significantly lower odds of receiving neuraxial anesthesia compared to White women, with aORs of 0.71 and 0.22, respectively. No significant differences were observed between non-Hispanic and Hispanic cohorts. The findings suggest the presence of racial disparities in anesthetic techniques for cesarean deliveries, emphasizing the need for a multidisciplinary approach to address these disparities despite the lack of disparity between Hispanic and non-Hispanic parturients [4].

Racial and ethnic disparities in access to local anesthesia for inguinal hernia repair: Of 78,766 patients aged 18 and older in the Veterans Affairs Surgical Quality Improvement Program database who underwent elective, unilateral, open inguinal hernia repair from 1998 to 2018, 23% of patients received local anesthesia, with Caucasians more frequently receiving local anesthesia (24%) compared to African Americans (17%) and Hispanics (19%). After adjusting for covariates, African Americans and Hispanics were significantly less likely to have hernia surgery under local anesthesia compared to Caucasians. Local anesthesia was associated with fewer postoperative complications for African American patients. While local anesthesia can enhance recovery, African Americans and Hispanics are less likely to receive it, highlighting the need for a better understanding of factors influencing anesthesia modality choices to address this disparity [7].

Racial and ethnic disparities in the mode of anesthesia for cesarean delivery: This study included 50,974 women across 19 US obstetric centers, categorized as Caucasian, African American, Hispanic, and Non-Hispanic Others (NHOs) from the Maternal-Fetal Medicine Units Network Cesarean Registry, covering 1999 to 2002. General anesthesia rates were 5.2% for Caucasians, 11.3% for African Americans, 5.8% for Hispanics, and 6.6% for NHOs. After adjusting for various covariates, African Americans had significantly higher odds of receiving general anesthesia compared to Caucasians (aOR: 1.7). Hispanic and NHO women also had higher odds (aOR: 1.1 and 1.2, respectively). Sensitivity analysis confirmed these findings, suggesting persistent disparities. The study concludes that African American women were most likely to undergo general anesthesia for cesarean delivery compared to other racial/ethnic groups, although the current state of these disparities in obstetric practice remains uncertain [8].

Racial and ethnic disparities in receipt of general anesthesia for cesarean delivery: This study examined whether rates of general anesthesia use for cesarean delivery differ by race or ethnicity. In a cross-sectional study of 35,117 patients, 3.3% received general anesthesia. The rates were 2.5% for Asian patients, 5.0% for Black patients, 3.7% for Hispanic patients, 2.8% for non-Hispanic White patients, and 3.8% for other groups. Among the 19,933 patients in labor at the time of cesarean delivery, 82.1% had neuraxial labor analgesia in situ. In this subgroup, no significant racial or ethnic differences in general anesthesia use were found (p = 0.16), suggesting that neuraxial labor analgesia may reduce disparities in general anesthesia use for cesarean delivery [10].

Theme 2: The Impact of Structural Racism on Anesthesia Patient Care

Effect of the COVID-19 pandemic on racial disparities in neuraxial labor analgesia at a Texas level IV maternal care center: Joint testing of coefficient p-values found no significant interaction between race and year of delivery. Multivariate logistic regression revealed that Hispanic (OR: 0.555) and Black or African American patients (OR: 0.613) were less likely to receive labor epidural analgesia compared to White patients. Higher gestational age increased the likelihood of receiving labor epidural analgesia, while higher parity decreased it. The COVID-19 pandemic did not significantly influence whether patients received labor epidural analgesia nor did the pandemic exacerbate racial disparities in labor epidural analgesia at the hospital [6].

Racial and ethnic disparities in neuraxial labor analgesia: This study explored racial and ethnic disparities in the use and anticipation of neuraxial analgesia among laboring Hispanic, African American, and Caucasian women at a large urban academic hospital through face-to-face surveys and post-delivery records. Results showed a univariate association between race/ethnicity and both anticipated and actual use of neuraxial analgesia. However, after adjusting for confounders, the association between race/ethnicity and actual use was not significant. Hispanic women were less likely to anticipate using neuraxial analgesia compared to Caucasian women (aOR: 0.40), even after controlling for confounders. The study concludes that while anticipated use of neuraxial analgesia was lower among Hispanic women, actual use was similar across racial/ethnic groups after accounting for confounding factors [9].

Structural racism and use of labor neuraxial analgesia among non-Hispanic Black birthing people: This study examined the link between structural racism, measured by inequity ratios in education, unemployment, and incarceration, and labor neuraxial analgesia use among non-Hispanic Black and White birthing people using 2017 US natality data. Results from 1,740,716 birth certificates (22.8% Black) showed that the labor neuraxial analgesia rate decreased with higher terciles of the racism index for both Black and White people. For Black people, the adjusted odds of receiving neuraxial analgesia were 18.4% lower in the second tercile and 28.3% lower in the third tercile compared to the first. For White people, the decreases were 13.4% and 15.6%, respectively. The study concluded that higher structural racism is associated with significantly reduced odds of receiving labor neuraxial analgesia, especially among Black people [16].

Reducing disparities: regional anesthesia blocks for mastectomy with reconstruction within standardized regional anesthesia pathways: Of 4,213 patients undergoing mastectomy with immediate tissue expander reconstruction from 2017 to 2022, 91% accepted and 9% declined a nerve block. Univariate analyses showed that White, non-Hispanic, English-speaking patients with commercial insurance and those undergoing bilateral reconstruction had the lowest rates of block refusal. The refusal rate decreased from 12% in 2017 to 6% in 2022. Multivariable logistic regression revealed that older age, Hispanic ethnicity, Medicaid status, unilateral surgery, and earlier study years were associated with higher rates of block refusal. The study concluded that standardized preoperative regional anesthesia programs can reduce racial disparities, although differences in age, ethnicity, and insurance status indicate a need for enhanced preoperative education to address patient hesitancies [14].

Racial and ethnic disparities in severe maternal morbidity and anesthetic techniques for obstetric deliveries: a multi-state analysis, 2007-2014: This retrospective cohort study analyzed data from 6,879,332 parturients from California, Florida, New York, Maryland, and Kentucky (2007-2014) to evaluate racial and ethnic disparities in anesthesia techniques. Results indicated that Black women had higher odds of experiencing severe maternal morbidity compared to White women (aOR: 1.38) and were more likely to receive general anesthesia for cesarean delivery (aOR: 1.44) and no analgesia for vaginal delivery (aOR: 1.45). These findings underscore significant differences in outcomes and care between Black and White parturients [11].

Theme 3: Racial Disparities in Pain Management

Are there racial disparities in perioperative pain? A retrospective study of a gynecological surgery cohort: A single-center, retrospective analysis using chart reviews of 203 patients who underwent minimally invasive hysterectomy was conducted. Three patients were excluded due to the small size of their racial cohorts, leaving 103 White and 100 Black patients for comparison. The study found no significant differences between these groups in postoperative pain scores in the post-anesthesia care unit or in the intraoperative analgesia administered by anesthesia providers. The results suggest no racial disparities in this specific population [5].

Racial disparities in operative procedure time: the influence of obesity: Using data from 47 hospitals in Illinois, New York, and Texas on elder Medicare patients (779 Black and 14,596 White) undergoing various surgeries between 2002 and 2006, the study matched Black patients to comparable White patients. Results showed that the mean BMI was higher in Black patients (30.24 kg/m²) than in White patients (28.96 kg/m²). After matching multiple factors, including BMI, the typical difference in anesthesia procedure time was seven minutes longer for Black patients, primarily due to a six-minute difference in surgical time. When procedure times differed by more than 30 minutes, Black patients more commonly had longer times. The study concludes that elder Black Medicare patients experience slightly longer procedure lengths than matched White controls, mainly due to differences in surgical time [12].

Racial disparities in the use of peripheral nerve blocks (PNBs) for postoperative analgesia after total mastectomy: a retrospective cohort study: This retrospective cohort study analyzed racial and ethnic disparities in the use of PNBs for postoperative analgesia in 64,103 patients undergoing total mastectomy under general anesthesia between 2015 and 2019. Results showed that only 7.3% of patients received a PNB. Non-Hispanic Asian and non-Hispanic Black patients had significantly reduced odds of receiving a PNB compared to non-Hispanic White patients, with odds ratios of 0.41 and 0.37, respectively. Non-Hispanic patients of other races, including American Indian, Alaskan Native, and Pacific Islander, also had reduced odds (OR: 0.73), as did Hispanic patients (OR: 0.62). The study found that PNBs were not associated with reduced odds of major complications after mastectomy (crude OR: 0.83; p = 0.17 and adjusted OR: 0.85; p = 0.21). These findings highlight significant disparities in PNB use across different racial and ethnic groups, emphasizing the need for efforts to ensure equitable access to postoperative analgesia [13].

Sensory nerve-mediated and nitric oxide-dependent cutaneous vasodilation in normotensive and prehypertensive non-Hispanic blacks and whites: 16 non-Hispanic Black and sixteen non-Hispanic White participants, divided into normotensive and prehypertensive groups (eight per subgroup), were tested using intradermal microdialysis fibers to measure skin blood flow. Sensory nerve-mediated vasodilation was reduced in prehypertensive non-Hispanic Whites (34 ± 7%) and both Black groups (normotensive, 20 ± 9%; prehypertensive, 24 ± 15%) compared to normotensive Whites (54 ± 12%). NO-dependent vasodilation was similarly reduced in prehypertensive Whites (41 ± 7%) and both Black groups (normotensive, 44 ± 7%; prehypertensive, 19 ± 7%) relative to normotensive Whites (60 ± 11%), with the greatest reduction in prehypertensive Blacks. These findings suggest that prehypertension adversely affects sensory-mediated and NO-dependent vasodilation in both racial groups, particularly in prehypertensive non-Hispanic Blacks [15].

The association of patient age, race, and demographic features on reported pain and sedation dosing during procedural abortion: a retrospective cohort study: A retrospective chart review of 225 patients from October 2019 to May 2020 using the Kruskal-Wallis H test showed no significant differences in fentanyl or midazolam dosing by age, with median doses of 75 mcg of fentanyl and 2 mg of midazolam across all age groups. However, White patients received higher median doses of midazolam than Black patients (3 mg vs. 2 mg, p < 0.01), despite similar pain scores. Additionally, patients terminating pregnancies for genetic anomalies received more fentanyl than those terminating for socioeconomic reasons (100 mcg vs. 75 mcg, p < 0.01). The study concludes that while age does not influence medication dosing, racial factors and reasons for abortion do, suggesting that demographic and psychosocial factors, as well as potential provider bias, affect both pain perception and analgesia dosage during abortion procedures [17].

The use of conscious sedation for pain control during forearm fracture reduction in children: does race matter?: Among a retrospective cohort study from a university-affiliated tertiary care children's hospital emergency department, of the 503 patients (83% Caucasian, 17% African American) undergoing closed reduction of a fractured ulna or radius over two years (excluding those with open reductions or multiple injuries), 404 received conscious sedation and 99 did not. Univariate analysis showed differences in fracture patterns (p = 0.0116) and angulation severity (p = 0.0094) between races. Multivariate analysis identified higher fracture translation (p < 0.0001), angulation (p < 0.0027), and younger age (p = 0.0059) as significant predictors of conscious sedation use, while race was not significantly associated (p = 0.0606 univariate, p = 0.1678 multivariate). The study concluded that the decision to use conscious sedation for pediatric forearm fractures was influenced by fracture characteristics and patient age, not race [18].

Discussion

Our review investigated the difference in treatment POC patients receive compared to Caucasian patients in the field of anesthesia. Our review found varying results ranging from significant racial disparities in the field to minimal racial disparities within the field. We found that when anesthesia is administered before a procedure on POC patients, they are likely to experience some form of racial mistreatment, such as not receiving anesthesia for a procedure. For example, one study showed that POC was less likely to receive anesthesia during an inguinal hernia repair surgery [7]. The study explained how this also led to an increase in postoperative complications for these patients [7]. To further the literature on this issue, it is important to address the factors as to why POC did not receive preoperative treatment and how this can be prevented for future operations.

Other studies reported no significant differences in anesthesia administration between POC and their Caucasian counterparts. For example, one study found that when POC patients and Caucasian patients underwent a minimally invasive hysterectomy, no significant differences were found in the pain scores during and after the operation [5]. Although there is some variation in results, many studies we reviewed still found significant racial disparities in the field of anesthesia, indicating that there is some racial basis when it comes to the treatment of POC patients. Future research should be conducted on the factors that contribute to the difference in care patients receive within anesthesia and healthcare. It is important to research how factors such as implicit biases and sociodemographic factors contribute to patient care discrepancies. There should also be future research done on specific anesthesiology procedures and how they are or are not impacted by racial disparities.

## Conclusions

Our review has concluded that when it comes to racial disparities and the impacts on health outcomes, the supporting evidence varies. Some studies suggest disparities, while others do not. Regardless, most studies we reviewed did find some racial disparities in the treatment of POC patients when receiving analgesics. Thus, it is important to explore these disparities and attempt to minimize them; doing so will not only provide better quality patient care but also improve the health outcomes of all patient populations. In addition, further research on the racial disparities in medicine and anesthesia is required to be conducted to definitively determine how racial disparities and biases impact the field of anesthesia. By determining how the disparities and biases impact the field, we can create and implement strategies that help combat these inequities and improve healthcare overall.

## Appendices

Appendix A

**Table 2:** Databases and corresponding search strings used for the literature search

Database	Search strings
Ovid Medline	1 ("people of color" or "people of colour" or "persons of color" or "persons of colour" or "person of color" or "person of colour" or "adult* of color" or "adult* of colour*").ti,ab. (2190) 2 exp Race Factors/ (1326) 3 ((race or racial) adj2 factor*).ti,ab. (2027) 4 (ethnicit* adj2 factor*).ti,ab. (863) 5 exp Minority Groups/ (18824) 6 exp "Black or African American"/ or exp Black People/ or (negro* or black* or "african american*").ti,ab. (298856) 7 exp "Hispanic or Latino"/ or exp Mexican Americans/ or (Hispanic* or hispano* or hispana* or Latino* or Latina* or Latine* or Latinx or "Latin X" or "latin american*" or "Spanish American*" or "Mexican American*" or Chicana* or Chicano* or Chicanx or "Chican x" or "Puerto Rican*" or Boricua* or "Cuban American*").ti,ab. (111751) 8 exp "American Indian or Alaska Native"/ or exp Indians, North American/ or ("native american*" or "american indian*" or "alaska native*" or indigenous).ti,ab. (72744) 9 exp Native Hawaiian/ or Other Pacific Islander/ or ("native hawaiian*" or "pacific islander*").ti,ab. (17593) 10 1 or 2 or 3 or 4 or 5 or 6 or 7 or 8 or 9 (449656) 11 exp PAIN PERCEPTION/ or exp PAIN MEASUREMENT/ or exp PAIN, PROCEDURAL/ or exp PAIN/ or exp PAIN THRESHOLD/ or exp PAIN MANAGEMENT/ (523074) 12 pain.ti,ab. (808657) 13 11 or 12 (994802) 14 10 and 13 (9421) 15 exp Bias, Implicit/ (195) 16 exp Stereotyping/ (12279) 17 (bias or biased or biases or stereotyp* or prejudice*).ti,ab. (349936) 18 exp Racism/ (7306) 19 exp Race Factors/ (1326) 20 ((race or racial) adj2 (factor* or discriminat* or disparit*)).ti,ab. (16540) 21 (ethnicit* adj2 (factor* or discriminat* or disparit*)).ti,ab. (1236) 22 (racism or racist*).ti,ab. (9888) 23 15 or 16 or 17 or 18 or 19 or 20 or 21 or 22 (384792) 24 1 or 5 or 6 or 7 or 8 or 9 (447861) 25 13 and 23 and 24 (742) 26 limit 25 to english language (739) 27 exp Analgesia/ (50458) 28 exp Analgesics/ (603385) 29 analges*.ti,ab. (149952) 30 exp Anesthesia, General/ (62925) 31 exp Anesthetics, General/ (139398) 32 (general adj5 (anesthetic* or anaesthetic* or anesthesia* or anaesthesia*)).mp. (95600) 33 (inhalation anesthetic* or inhalation anaesthetic* or anesthetic gases or anaesthetic gases or anesthetic gas or anaesthetic gas).mp. (2893) 34 (intravenous anesthetic* or intravenous anaesthetic*).mp. (2116) 35 (dissociative anesthesia* or dissociative anaesthesia* or dissociative anesthetic* or dissociative anaesthetic*).mp. (572) 36 (Desflurane or suprane).mp. (2771) 37 (Enflurane or alyrane or enfran or enlirane or ethrane or etran).mp. (3735) 38 ether.mp. (66890) 39 (halothane or fluothane or ftorotan or narcotan).mp. (19824) 40 isoflurane.mp. (15928) 41 (Methoxyflurane or anecotan or methofluranum or penthrane or pentrane).mp. (2427) 42 (nitrous oxide or "laughing gas" or nitrogen protoxide).mp. (24780) 43 (Sevoflurane or sevorane or ultane or "fluoromethyl hexafluoroisopropyl ether").mp. (11541) 44 (Trichloroethylene or ethinyl trichloride or trichloroethene or trielina or trilene).mp. (6490) 45 xenon.mp. (12820) 46 (Alfentanil or alfenta or alfentanyl or fanaxal or limifen or rapifen).mp. (2548) 47 (Chloralose or anhydroglucochloral or glucochloral or glucochloralose).mp. (5557) 48 (Diazepam or apaurin or diazemuls or faustan or relanium or seduxen or sibazon or stesolid or valium).mp. (27437) 49 (Etomidate or ethomidate or hypnomidate or radenarkon).mp. (3157) 50 (Fentanyl or duragesic or durogesic or fentanest or fentora or mun5lyg46h or phentanyl or sublimaze).mp. (26783) 51 (Methohexital or brevimytal natrium or brevital or brietal or methohexitone).mp. (1905) 52 (Midazolam or dormicum or midazolam or versed).mp. (18053) 53 (Propanidid or epontol or panitol or sombrevin).mp. (1065) 54 (Propofol* or aquafol or diprivan or disoprivan or disoprofol or fresofol or ivofol or recofol).mp. (26842) 55 (Sodium Oxybate or sodium oxybutyrate or somsanit or xyrem or gamma hydroxybutyrate).mp. (2777) 56 (Sufentanil or sufenta or sulfentanil or sulfentanyl).mp. (3790) 57 (Thiamylal or surital or thioquinalbarbitone).mp. (797) 58 (Thiopental or bomathal or nesdonal or penthiobarbital or pentothal or sodipental or thiomebumal or thionembutal or thiopentobarbital or thiopentone or tiobarbital braun or trapanal).mp. (10673) 59 (Urethane or ethyl carbamate or urethan).mp. (16275) 60 (Ketamine or calipsol or calypsol or kalipsol or ketalar or ketanest or ketaset).mp. (25341) 61 Tiletamine.mp. (526) 62 30 or 31 or 32 or 33 or 34 or 35 or 36 or 37 or 38 or 39 or 40 or 41 or 42 or 43 or 44 or 45 or 46 or 47 or 48 or 49 or 50 or 51 or 52 or 53 or 54 or 55 or 56 or 57 or 58 or 59 or 60 or 61 (356867) 63 anesthesia, conduction/ or anesthesia, epidural/ or anesthesia, caudal/ or anesthesia, local/ or anesthesia, spinal/ or nerve block/ or autonomic nerve block/ or sphenopalatine ganglion block/ or brachial plexus block/ or cervical plexus block/ (73105) 64 exp Anesthetics, Local/ (113069) 65 ((local or regional or "loco‐regional" or locoregional) adj5 (anesthetic* or anaesthetic* or anesthesia* or anaesthesia*)).mp. (91838) 66 ((conduction or peridural* or epidural* or caudal or infiltration or spinal) adj5 (anesthetic* or anaesthetic* or anesthesia* or anaesthesia*)).mp. (48377) 67 ("Neural Therapy of Huneke" or "nerve block*" or "Chemical Neurolys*" or Chemodenervation* or "Sphenopalatine Ganglion Block*" or "Pterygopalatine Ganglion Block*" or "Brachial Plexus Block*" or "Brachial Plexus Anesthesia*" or "Brachial Plexus Anaesthesia*" or "Cervical Plexus Block*" or "Cervical Plexus Anesthesia*" or "Cervical Plexus Anaesthesia*" or ((nerv* or neuraxial) adj3 block*)).mp. (38772) 68 (paravertebral* adj5 (anesthetic* or anaesthetic* or anesthesia* or anaesthesia* or analgesi* or block*)).mp. (1956) 69 (benzocaine or americaine or anaesthesin or anesthesin or bensokain or ethoform or "ethyl aminobenzoate" or "4-aminobenzoic acid ethyl ester").mp. (1936) 70 (Bupivacaine or bupivacain or buvacaina or carbostesin ordolanaest or marcain or marcaine or sensorcaine or "svedocain sin vasoconstr").mp. (18931) 71 (Carticaine or articain or articaine or carticain or ultracaine).mp. (935) 72 (Dibucaine or cincain or cinchocaine or nupercainal or nupercaine or sovcaine).mp. (1513) 73 (Etidocaine or duranest).mp. (395) 74 (Levobupivacaine or chirocaine).mp. (1705) 75 (Lidocaine or dalcaine or lignocaine or octocaine or xylesthesin or xylocaine or xylocitin or xyloneural).mp. (37430) 76 (Mepivacaine or carbocaine or isocaine or isogaine or meaverin or mecain or mepihexal or mepivacain or mepivastesin or polocaine or scandicain or scandicaine or scandinibsa or scandonest).mp. (2858) 77 (Prilocaine or citanest or propitocaine or xylonest).mp. (2865) 78 (Propoxycaine or propoxyprocaine).mp. (287) 79 (Ropivacaine or naropeine or naropin or ropivacaine).mp. (6574) 80 (Tetracaine or amethocaine or ametop or dicaine or pantocaine or pontocaine or tetrakain).mp. (3568) 81 (Trimecaine or mesocaine).mp. (372) 82 63 or 64 or 65 or 66 or 67 or 68 or 69 or 70 or 71 or 72 or 73 or 74 or 75 or 76 or 77 or 78 or 79 or 80 or 81 (215814) 83 exp Anesthesiology/ (33780) 84 exp Anesthesiologists/ (2103) 85 (anesthesiolog* or anesthetist* or anaesthesiolog* or anaesthetist*).ti,ab. (56301) 86 (Adenosine or Clonidine or Duloxetine or enkephalin or Flurbiprofen or Gabapentin or Ketamine or Magnesium Sulfate or Mitoxantrone or Pregabalin or Xylazine or Acetaminophen or benzeneacetamide or Amantadine or Amitriptyline or Carbachol or Carbamazepine or Cyclazocine or Dexmedetomidine or Dihydroergotamine or Dronabinol or Ergotamine or Glafenine or Ibuprofen or "Interleukin-2" or Interleukin2 or Medetomidine or Methotrimeprazine or Milnacipran or Nefopam or "Nitrous Oxide" or Phenacetin or Pizotyline or Quinine or BW755C or "BW 755C" or Adapalene or Ampyrone or Antipyrine or Apazone or Aspirin or Bufexamac or Celecoxib or Clonixin or Curcumin or Diclofenac or Diflunisal or Dipyrone or Epirizole or Etanercept or Etodolac or Etoricoxib or Fenoprofen or Feprazone or Flurbiprofen or Ibuprofen or Indomethacin or Ketoprofen or Ketorolac or Meclofenamic or Mefenamic or Meloxicam or Mesalamine or Nabumetone or Naproxen or "Niflumic Acid" or Olopatadine or Oxaprozin or Oxyphenbutazone or Phenylbutazone or Piroxicam or Salicylate* or "Salicylic Acid" or "Sodium Salicylate" or "Acetylsalicylic acid" or Sulfasalazine or Sulindac or Suprofen or Tolmetin or Alfentanil or Alphaprodine or Buprenorphine or Butorphanol or Codeine or Dextromoramide or Dextropropoxyphene or Dihydromorphine or Ethylmorphine or Etorphine or Fentanyl or Heroin or Hydrocodone or Hydromorphone or Levorphanol or Meperidine or Meptazinol or Methadone or Morphine or Opiate* or Opium or Opioid* or Oxycodone or Oxymorphone or Pentazocine or Phenazocine or Phenoperidine or Pirinitramide or Promedol or Sufentanil or Thebaine or Tilidine or Tramadol or Diphenoxylate or Enkephalin or Ethylketocyclazocine or Ethylmorphine or Etorphine or "Methadyl Acetate" or Nalbuphine or Remifentanil or Tapentadol).ti,ab. (726518) 87 27 or 28 or 29 or 62 or 82 or 83 or 84 or 85 or 86 (1459517) 88 23 and 24 and 87 (537) 89 limit 88 to english language (535) 90 26 or 89 (1103)
Cochrane	ID Search Hits #1 ("people of color" or "people of colour" or "persons of color" or "persons of colour" or "person of color" or "person of colour" or "adult of color" or "adult of colour" or "adults of color" or "adults of colour"):ti,ab 100 #2 MeSH descriptor: [Minority Groups] explode all trees 609 #3 MeSH descriptor: [Black or African American] explode all trees 3148 #4 MeSH descriptor: [Black People] explode all trees 4148 #5 (negro* or black* or "african american" or "african americans"):ti,ab 19553 #6 MeSH descriptor: [Hispanic or Latino] explode all trees 2136 #7 MeSH descriptor: [Mexican Americans] explode all trees 203 #8 (Hispanic* or hispano* or hispana* or latino or latina or latinos or latinas or "latin x" or latinx or "latin american" or "latin americans" or "Spanish American" or "Spanish Americans" or "Spanish American" or "Spanish Americans" or "Mexican American" or "Mexican Americans" or Chicana* or Chicano* or Chicanx or "Chican x" or "Puerto Rican" or "Puerto Ricans" or "Cuban American" or "Cuban Americans" or Buricua*):ti,ab 10004 #9 (Boricua*):ti,ab 2 #10 MeSH descriptor: [American Indian or Alaska Native] explode all trees 472 #11 MeSH descriptor: [Indians, North American] explode all trees 400 #12 ("native american" or "native americans" or "american indian" or "american indians" or "alaska native" or "alaska natives" or indigenous):ti,ab 2011 #13 #1 or #2 or #3 or #4 or #5 or #6 or #7 or #8 or #9 or #10 or #11 or #12 28318 #14 MeSH descriptor: [Bias, Implicit] explode all trees 4 #15 MeSH descriptor: [Stereotyping] explode all trees 534 #16 MeSH descriptor: [Racism] explode all trees 91 #17 MeSH descriptor: [Race Factors] explode all trees 49 #18 (bias or biased or biases or stereotyp* or prejudice*):ti,ab 24308 #19 ((race or racial) near/2 (factor* or discriminat* or disparit*)):ti,ab 790 #20 ((ethnicit*) near/2 (factor* or discriminat* or disparit*)):ti,ab 50 #21 (racism or racist*):ti,ab 180 #22 #14 or #15 or #16 or #17 or #18 or #19 or #20 or #21 25491 #23 MeSH descriptor: [Pain Perception] explode all trees 776 #24 MeSH descriptor: [Pain Measurement] explode all trees 26855 #25 MeSH descriptor: [undefined] explode all trees 0 #26 MeSH descriptor: [Pain] explode all trees 72505 #27 MeSH descriptor: [Pain Threshold] explode all trees 2240 #28 MeSH descriptor: [Pain Management] explode all trees 5955 #29 pain:ti,ab 225290 #30 #23 or #24 or #25 or #26 or #27 or #28 or #29 241442 #31 #13 and #22 and #30 162 #32 MeSH descriptor: [Analgesia] explode all trees 10637 #33 MeSH descriptor: [Analgesics] explode all trees 27937 #34 MeSH descriptor: [Anesthesia, General] explode all trees 8351 #35 MeSH descriptor: [Anesthetics, General] explode all trees 6330 #36 MeSH descriptor: [Anesthetics, Local] explode all trees 10808 #37 MeSH descriptor: [Anesthesia, Conduction] explode all trees 12850 #38 MeSH descriptor: [Anesthesia, Epidural] explode all trees 2320 #39 MeSH descriptor: [Anesthesia, Caudal] explode all trees 262 #40 MeSH descriptor: [Anesthesia, Local] explode all trees 2739 #41 MeSH descriptor: [Anesthesia, Spinal] explode all trees 2908 #42 MeSH descriptor: [Nerve Block] explode all trees 5824 #43 MeSH descriptor: [Autonomic Nerve Block] explode all trees 345 #44 MeSH descriptor: [Sphenopalatine Ganglion Block] explode all trees 28 #45 MeSH descriptor: [Brachial Plexus Block] explode all trees 340 #46 MeSH descriptor: [Cervical Plexus Block] explode all trees 42 #47 MeSH descriptor: [Anesthesiology] explode all trees 584 #48 MeSH descriptor: [Anesthesiologists] explode all trees 81 #49 (analges* or anesthesiolog* or anesthetist* or anaesthesiolog* or anaesthetist*):ti,ab 85253 #50 (general near/5 (anesthetic* or anaesthetic* or anesthesia* or anaesthesia*)):ti,ab 30024 #51 ((local or regional or "loco‐regional" or locoregional) near/5 (anesthetic* or anaesthetic* or anesthesia* or anaesthesia*)):ti,ab 23234 #52 ((conduction or peridural* or epidural* or caudal or infiltration or spinal) near/5 (anesthetic* or anaesthetic* or anesthesia* or anaesthesia*)):ti,ab 18359 #53 ("Neural Therapy of Huneke" or "nerve block" or "nerve blocks" or "Chemical Neurolyses" or "Chemical Neurolysis" or Chemodenervation* or "Sphenopalatine Ganglion Block" or "Sphenopalatine Ganglion Blocks" or "Pterygopalatine Ganglion Block" or "Pterygopalatine Ganglion Blocks" or "Brachial Plexus Block" or "Brachial Plexus Blocks" or "Brachial Plexus Anesthesia" or "Brachial Plexus Anaesthesia" or "Cervical Plexus Block" or "Cervical Plexus Blocks" or "Cervical Plexus Anesthesia" or "Cervical Plexus Anaesthesia"):ti,ab 11679 #54 (paravertebral* near/5 (anesthetic* or anaesthetic* or anesthesia* or anaesthesia* or analgesi* or block*)):ti,ab 1803 #55 (benzocaine or americaine or anaesthesin or anesthesin or bensokain or ethoform or "ethyl aminobenzoate" or "4-aminobenzoic acid ethyl ester"):ti,ab 300 #56 (Bupivacaine or bupivacain or buvacaina or carbostesin ordolanaest or marcain or marcaine or sensorcaine or "svedocain sin vasoconstr"):ti,ab 15583 #57 (Carticaine or articain or articaine or carticain or ultracaine):ti,ab 803 #58 (Dibucaine or cincain or cinchocaine or nupercainal or nupercaine or sovcaine):ti,ab 42 #59 (Etidocaine or duranest or Levobupivacaine or chirocaine or Lidocaine or dalcaine or lignocaine or octocaine or xylesthesin or xylocaine or xylocitin or xyloneural or Mepivacaine or carbocaine or isocaine or isogaine or meaverin or mecain or mepihexal or mepivacain or mepivastesin or polocaine or scandicain or scandicaine or scandinibsa or scandonest or Prilocaine or citanest or propitocaine or xylonest or Propoxycaine or propoxyprocaine or Ropivacaine or naropeine or naropin or ropivacaine or Tetracaine or amethocaine or ametop or dicaine or pantocaine or pontocaine or tetrakain or Trimecaine or mesocaine):ti,ab 27967 #60 (Adenosine or Clonidine or Duloxetine or enkephalin or Flurbiprofen or Gabapentin or Ketamine or Magnesium Sulfate or Mitoxantrone or Pregabalin or Xylazine or Acetaminophen or benzeneacetamide or Amantadine or Amitriptyline or Carbachol or Carbamazepine or Cyclazocine or Dexmedetomidine or Dihydroergotamine or Dronabinol or Ergotamine or Glafenine or Ibuprofen or "Interleukin-2" or Interleukin2 or Medetomidine or Methotrimeprazine or Milnacipran or Nefopam or "Nitrous Oxide" or Phenacetin or Pizotyline or Quinine or BW755C or "BW 755C" or Adapalene or Ampyrone or Antipyrine or Apazone or Aspirin or Bufexamac or Celecoxib or Clonixin or Curcumin or Diclofenac or Diflunisal or Dipyrone or Epirizole or Etanercept or Etodolac or Etoricoxib or Fenoprofen or Feprazone or Flurbiprofen or Ibuprofen or Indomethacin or Ketoprofen or Ketorolac or Meclofenamic or Mefenamic or Meloxicam or Mesalamine or Nabumetone or Naproxen or "Niflumic Acid" or Olopatadine or Oxaprozin or Oxyphenbutazone or Phenylbutazone or Piroxicam or Salicylate* or "Salicylic Acid" or "Sodium Salicylate" or "Acetylsalicylic acid" or Sulfasalazine or Sulindac or Suprofen or Tolmetin or Alfentanil or Alphaprodine or Buprenorphine or Butorphanol or Codeine or Dextromoramide or Dextropropoxyphene or Dihydromorphine or Ethylmorphine or Etorphine or Fentanyl or Heroin or Hydrocodone or Hydromorphone or Levorphanol or Meperidine or Meptazinol or Methadone or Morphine or Opiate* or Opium or Opioid* or Oxycodone or Oxymorphone or Pentazocine or Phenazocine or Phenoperidine or Pirinitramide or Promedol or Sufentanil or Thebaine or Tilidine or Tramadol or Diphenoxylate or Enkephalin or Ethylketocyclazocine or Ethylmorphine or Etorphine or "Methadyl Acetate" or Nalbuphine or Remifentanil or Tapentadol):ti,ab 145938 #61 ("inhalation anesthetic" or "inhalation anesthetics" or "inhalation anaesthetic" or "inhalation anaesthetics" or "anesthetic gases" or "anaesthetic gases" or "anesthetic gas" or "anaesthetic gas"):ti,ab 520 #62 ("intravenous anesthetic" or "intravenous anaesthetic" or "intravenous anesthetics" or "intravenous anaesthetics"):ti,ab 548 #63 ("dissociative anesthesia" or "dissociative anaesthesia" or "dissociative anesthetic" or "dissociative anaesthetic" or "dissociative anesthesias" or "dissociative anaesthesias" or "dissociative anesthetics" or "dissociative anaesthetics"):ti,ab 63 #64 (Desflurane or suprane or Enflurane or alyrane or enfran or enlirane or ethrane or etran or ether or halothane or fluothane or ftorotan or narcotan or isoflurane or Methoxyflurane or anecotan or methofluranum or penthrane or pentrane or "nitrous oxide" or "laughing gas" or "nitrogen protoxide" or Sevoflurane or sevorane or ultane or "fluoromethyl hexafluoroisopropyl ether" or Trichloroethylene or ethinyl trichloride or trichloroethene or trielina or trilene or xenon or Alfentanil or alfenta or alfentanyl or fanaxal or limifen or rapifen or Chloralose or anhydroglucochloral or glucochloral or glucochloralose or Diazepam or apaurin or diazemuls or faustan or relanium or seduxen or sibazon or stesolid or valium or Etomidate or ethomidate or hypnomidate or radenarkon or Fentanyl or duragesic or durogesic or fentanest or fentora or mun5lyg46h or phentanyl or sublimaze or Methohexital or brevimytal natrium or brevital or brietal or methohexitone or Midazolam or dormicum or midazolam or versed or Propanidid or epontol or panitol or sombrevin or Propofol* or aquafol or diprivan or disoprivan or disoprofol or fresofol or ivofol or recofol or "Sodium Oxybate" or "sodium oxybutyrate" or somsanit or xyrem or "gamma hydroxybutyrate" or Sufentanil or sufenta or sulfentanil or sulfentanyl or Thiamylal or surital or thioquinalbarbitone or Thiopental or bomathal or nesdonal or penthiobarbital or pentothal or sodipental or thiomebumal or thionembutal or thiopentobarbital or thiopentone or tiobarbital braun or trapanal or Urethane or ethyl carbamate or urethan or Ketamine or calipsol or calypsol or kalipsol or ketalar or ketanest or ketaset or Tiletamine):ti,ab53763 #65 #32 or #33 or #34 or #35 or #36 or #37 or #38 or #39 or #40 or #41 or #42 or #43 or #44 o #45 or #46 or #47 or #48 or #49 or #50 or #51 or #52 or #53 or #54 or #55 or #56 o #57 or #58 or #59 or #60 or #61 or #62 or #63 or #64 237099 #66 #13 and #22 and #65 93 #67 #31 or #66 211
Scopus	( ( ( TITLE-ABS ( "people of color" OR "people of colour" OR "persons of color" OR "persons of colour" OR "person of color" OR "person of colour" OR "adult of color" OR "adult of colour" OR "adults of color" OR "adults of colour" ) ) OR ( TITLE-ABS ( negro* OR black* OR "african american" OR "african americans" OR hispanic* OR hispano* OR hispana* OR latino OR latina OR latinos OR latinas OR "latin x" OR latinx OR "latin american" OR "latin americans" OR "Spanish American" OR "Spanish Americans" OR "Spanish American" OR "Spanish Americans" OR "Mexican American" OR "Mexican Americans" OR chicana* OR chicano* OR chicanx OR "Chican x" OR "Puerto Rican" OR "Puerto Ricans" OR "Cuban American" OR "Cuban Americans" OR buricua* OR "native american" OR "native americans" OR "american indian" OR "american indians" OR "alaska native" OR "alaska natives" OR indigenous ) ) ) AND ( ( TITLE-ABS ( ( race OR racial ) W/2 ( factor* OR discriminat* OR disparit* ) ) ) OR ( TITLE-ABS ( bias OR biased OR biases OR stereotyp* OR prejudice* ) ) OR ( TITLE-ABS ( ( ethnicit* ) W/2 ( factor* OR discriminat* OR disparit* ) ) ) OR ( TITLE-ABS ( racism OR racist* ) ) ) AND ( ( TITLE-ABS ( analges* OR anesthesiolog* OR anesthetist* OR anaesthesiolog* OR anaesthetist* ) ) OR ( TITLE-ABS ( general W/5 ( anesthetic* OR anaesthetic* OR anesthesia* OR anaesthesia* ) ) ) OR ( TITLE-ABS ( ( local OR regional OR "loco‐regional" OR locoregional ) W/5 ( anesthetic* OR anaesthetic* OR anesthesia* OR anaesthesia* ) ) ) OR ( TITLE-ABS ( ( conduction OR peridural* OR epidural* OR caudal OR infiltration OR spinal ) W/5 ( anesthetic* OR anaesthetic* OR anesthesia* OR anaesthesia* ) ) ) OR ( TITLE-ABS ( "Neural Therapy of Huneke" OR "nerve block" OR "nerve blocks" OR "Chemical Neurolyses" OR "Chemical Neurolysis" OR chemodenervation* OR "Sphenopalatine Ganglion Block" OR "Sphenopalatine Ganglion Blocks" OR "Pterygopalatine Ganglion Block" OR "Pterygopalatine Ganglion Blocks" OR "Brachial Plexus Block" OR "Brachial Plexus Blocks" OR "Brachial Plexus Anesthesia" OR "Brachial Plexus Anaesthesia" OR "Cervical Plexus Block" OR "Cervical Plexus Blocks" OR "Cervical Plexus Anesthesia" OR "Cervical Plexus Anaesthesia" ) ) OR ( TITLE-ABS ( paravertebral* W/5 ( anesthetic* OR anaesthetic* OR anesthesia* OR anaesthesia* OR analgesi* OR block* ) ) ) OR ( TITLE-ABS ( benzocaine OR americaine OR anaesthesin OR anesthesin OR bensokain OR ethoform OR "ethyl aminobenzoate" OR "4-aminobenzoic acid ethyl ester" OR bupivacaine OR bupivacain OR buvacaina OR carbostesin AND ordolanaest OR marcain OR marcaine OR sensorcaine OR "svedocain sin vasoconstr" OR carticaine OR articain OR articaine OR carticain OR ultracaine OR dibucaine OR cincain OR cinchocaine OR nupercainal OR nupercaine OR sovcaine OR etidocaine OR duranest OR levobupivacaine OR chirocaine OR lidocaine OR dalcaine OR lignocaine OR octocaine OR xylesthesin OR xylocaine OR xylocitin OR xyloneural OR mepivacaine OR carbocaine OR isocaine OR isogaine OR meaverin OR mecain OR mepihexal OR mepivacain AND ormepivastesin OR polocaine OR scandicain OR scandicaine OR scandinibsa OR scandonest OR prilocaine OR citanest OR propitocaine OR xylonest OR propoxycaine OR propoxyprocaine OR ropivacaine OR naropeine OR naropin OR ropivacaine OR tetracaine OR amethocaine OR ametop OR dicaine OR pantocaine OR pontocaine OR tetrakain OR trimecaine OR mesocaine ) ) OR ( TITLE-ABS ( adenosine OR clonidine OR duloxetine OR enkephalin OR flurbiprofen OR gabapentin OR ketamine OR "Magnesium Sulfate" OR mitoxantrone OR pregabalin OR xylazine OR acetaminophen OR benzeneacetamide OR amantadine OR amitriptyline OR carbachol OR carbamazepine OR cyclazocine OR dexmedetomidine OR dihydroergotamine OR dronabinol OR ergotamine OR glafenine OR ibuprofen OR "Interleukin-2" OR interleukin2 OR medetomidine OR methotrimeprazine OR milnacipran OR nefopam OR "Nitrous Oxide" OR phenacetin OR pizotyline OR quinine OR bw755c OR "BW 755C" OR adapalene OR ampyrone OR antipyrine OR apazone OR aspirin OR bufexamac OR celecoxib OR clonixin OR curcumin OR diclofenac OR diflunisal OR dipyrone OR epirizole OR etanercept OR etodolac OR etoricoxib OR fenoprofen OR feprazone OR flurbiprofen OR ibuprofen OR indomethacin OR ketoprofen OR ketorolac OR meclofenamic OR mefenamic OR meloxicam OR mesalamine OR nabumetone AND ornaproxen OR "Niflumic Acid" OR olopatadine OR oxaprozin OR oxyphenbutazone OR phenylbutazone OR piroxicam OR salicylate* OR "Salicylic Acid" OR "Sodium Salicylate" OR "Acetylsalicylic acid" OR sulfasalazine OR sulindac OR suprofen OR tolmetin OR alfentanil OR alphaprodine OR buprenorphine OR butorphanol OR codeine OR dextromoramide OR dextropropoxyphene OR dihydromorphine OR ethylmorphine OR etorphine OR fentanyl OR heroin OR hydrocodone OR hydromorphone OR levorphanol OR meperidine OR meptazinol OR methadone OR morphine OR opiate* OR opium OR opioid* OR oxycodone OR oxymorphone OR pentazocine OR phenazocine OR phenoperidine OR pirinitramide OR promedol OR sufentanil OR thebaine OR tilidine OR tramadol OR diphenoxylate OR enkephalin OR ethylketocyclazocine OR ethylmorphine OR etorphine OR "Methadyl Acetate" OR nalbuphine OR remifentanil OR tapentadol ) ) OR ( TITLE-ABS ( "inhalation anesthetic" OR "inhalation anesthetics" OR "inhalation anaesthetic" OR "inhalation anaesthetics" OR "anesthetic gases" OR "anaesthetic gases" OR "anesthetic gas" OR "anaesthetic gas" OR "intravenous anesthetic" OR "intravenous anaesthetic" OR "intravenous anesthetics" OR "intravenous anaesthetics" OR "dissociative anesthesia" OR "dissociative anaesthesia" OR "dissociative anesthetic" OR "dissociative anaesthetic" OR "dissociative anesthesias" OR "dissociative anaesthesias" OR "dissociative anesthetics" OR "dissociative anaesthetics" ) ) OR ( TITLE-ABS ( desflurane OR suprane OR enflurane OR alyrane OR enfran OR enlirane OR ethrane OR etran OR ether OR halothane OR fluothane OR ftorotan OR narcotan OR isoflurane OR methoxyflurane OR anecotan OR methofluranum OR penthrane OR pentrane OR "nitrous oxide" OR "laughing gas" OR "nitrogen protoxide" OR sevoflurane OR sevorane OR ultane OR "fluoromethyl hexafluoroisopropyl ether" OR trichloroethylene OR "ethinyl trichloride" OR trichloroethene OR trielina OR trilene OR xenon OR alfentanil OR alfenta OR alfentanyl OR fanaxal OR limifen OR rapifen ) ) OR ( TITLE-ABS ( diazepam OR apaurin OR diazemuls OR faustan OR relanium OR seduxen OR sibazon OR stesolid OR valium OR etomidate OR ethomidate OR hypnomidate OR radenarkon OR fentanyl OR duragesic OR durogesic OR fentanest OR fentora OR "mun5lyg46h" OR phentanyl OR sublimaze ) ) OR ( TITLE-ABS ( chloralose OR anhydroglucochloral OR glucochloral OR glucochloralose ) ) OR ( TITLE-ABS ( propofol* OR aquafol OR diprivan OR disoprivan OR disoprofol OR fresofol OR ivofol OR recofol ) ) OR ( TITLE-ABS ( midazolam OR dormicum OR midazolam OR versed ) ) OR ( TITLE-ABS ( propanidid OR epontol OR panitol OR sombrevin ) ) OR ( TITLE-ABS ( methohexital OR "brevimytal natrium" OR brevital OR brietal OR methohexitone ) ) OR ( TITLE-ABS ( "Sodium Oxybate" OR "sodium oxybutyrate" OR somsanit OR xyrem OR "gamma hydroxybutyrate" ) ) OR ( TITLE-ABS ( sufentanil OR sufenta OR sulfentanil OR sulfentanyl ) ) OR ( TITLE-ABS ( thiamylal OR surital OR thioquinalbarbitone ) ) OR ( TITLE-ABS ( thiopental OR bomathal OR nesdonal OR penthiobarbital OR pentothal OR sodipental OR thiomebumal OR thionembutal OR thiopentobarbital OR thiopentone OR "tiobarbital braun" OR trapanal ) ) OR ( TITLE-ABS ( urethane OR ethyl AND carbamate OR urethan ) ) OR ( TITLE-ABS ( ketamine OR calipsol OR calypsol OR kalipsol OR ketalar OR ketanest OR ketaset ) ) OR ( TITLE-ABS ( tiletamine ) ) ) ) OR ( ( ( TITLE-ABS ( "people of color" OR "people of colour" OR "persons of color" OR "persons of colour" OR "person of color" OR "person of colour" OR "adult of color" OR "adult of colour" OR "adults of color" OR "adults of colour" ) ) OR ( TITLE-ABS ( negro* OR black* OR "african american" OR "african americans" OR hispanic* OR hispano* OR hispana* OR latino OR latina OR latinos OR latinas OR "latin x" OR latinx OR "latin american" OR "latin americans" OR "Spanish American" OR "Spanish Americans" OR "Spanish American" OR "Spanish Americans" OR "Mexican American" OR "Mexican Americans" OR chicana* OR chicano* OR chicanx OR "Chican x" OR "Puerto Rican" OR "Puerto Ricans" OR "Cuban American" OR "Cuban Americans" OR buricua* OR "native american" OR "native americans" OR "american indian" OR "american indians" OR "alaska native" OR "alaska natives" OR indigenous ) ) ) AND ( ( TITLE-ABS ( ( race OR racial ) W/2 ( factor* OR discriminat* OR disparit* ) ) ) OR ( TITLE-ABS ( bias OR biased OR biases OR stereotyp* OR prejudice* ) ) OR ( TITLE-ABS ( ( ethnicit* ) W/2 ( factor* OR discriminat* OR disparit* ) ) ) OR ( TITLE-ABS ( racism OR racist* ) ) ) AND ( TITLE-ABS ( pain ) ) ) AND ( LIMIT-TO ( LANGUAGE , "English" ) )
